# The Effects of Artificial Intelligence Assistance on the Radiologists’ Assessment of Lung Nodules on CT Scans: A Systematic Review

**DOI:** 10.3390/jcm12103536

**Published:** 2023-05-18

**Authors:** Lotte J. S. Ewals, Kasper van der Wulp, Ben E. E. M. van den Borne, Jon R. Pluyter, Igor Jacobs, Dimitrios Mavroeidis, Fons van der Sommen, Joost Nederend

**Affiliations:** 1 Department of Radiology, Catharina Cancer Institute, Catharina Hospital Eindhoven, 5623 EJ Eindhoven, The Netherlands; 2 Department of Electrical Engineering, Eindhoven University of Technology, 5612 AZ Eindhoven, The Netherlands; 3 Department of Pulmonology, Catharina Cancer Institute, Catharina Hospital Eindhoven, 5623 EJ Eindhoven, The Netherlands; 4 Department of Experience Design, Royal Philips, 5656 AE Eindhoven, The Netherlands; 5 Department of Hospital Services and Informatics, Philips Research, 5656 AE Eindhoven, The Netherlands; 6 Department of Data Science, Philips Research, 5656 AE Eindhoven, The Netherlands

**Keywords:** artificial intelligence, diagnosis, computer-assisted, detection, lung neoplasms, malignancy prediction, systematic review, tomography, X-ray computed

## Abstract

To reduce the number of missed or misdiagnosed lung nodules on CT scans by radiologists, many Artificial Intelligence (AI) algorithms have been developed. Some algorithms are currently being implemented in clinical practice, but the question is whether radiologists and patients really benefit from the use of these novel tools. This study aimed to review how AI assistance for lung nodule assessment on CT scans affects the performances of radiologists. We searched for studies that evaluated radiologists’ performances in the detection or malignancy prediction of lung nodules with and without AI assistance. Concerning detection, radiologists achieved with AI assistance a higher sensitivity and AUC, while the specificity was slightly lower. Concerning malignancy prediction, radiologists achieved with AI assistance generally a higher sensitivity, specificity and AUC. The radiologists’ workflows of using the AI assistance were often only described in limited detail in the papers. As recent studies showed improved performances of radiologists with AI assistance, AI assistance for lung nodule assessment holds great promise. To achieve added value of AI tools for lung nodule assessment in clinical practice, more research is required on the clinical validation of AI tools, impact on follow-up recommendations and ways of using AI tools.

## 1. Introduction

Because of a lack of symptoms in early stages, lung cancer is often discovered in an advanced stage. These late diagnoses limit curative treatment options and thereby worsen the prognosis for patients [1]. Consequently, lung cancer is associated with a high mortality rate. Annually, there are approximately 2.2 million new diagnoses and about 1.8 million deaths worldwide [2]. To improve survival rates, early detection is a major focus in lung cancer research. The importance of early detection, amongst others, was demonstrated by the National Lung Screening Trial (NLST). This study evaluated the effects of performing lung cancer screening using low-dose Computed Tomography (CT) in high-risk patients with a history of smoking. In 24.2% of the cases, the screening result was positive; however, 96.4% of these positive findings appeared to be a benign finding. Nevertheless, screening reduced the mortality by 20% [3]. Furthermore, on CT scans of the lungs, actionable nodules, which are the nodules that require any form of follow-up, can be missed or misinterpreted by radiologists. This may lead to a delayed diagnosis and, consequently, a delayed start of treatment. Therefore, it is important to aid radiologists in their assessments of lung nodules on CT scans.

To provide patients with more appropriate follow-up advice, to improve the consistency of diagnoses and to reduce the workload of radiologists, numerous studies have explored the use of Artificial Intelligence (AI) for lung nodule assessment on CT scans. These studies focused on several aspects of the assessment, including the detection of lung nodules and the distinction between malignant and benign nodules. In recent years, the AI algorithms for the assessment of lung nodules have been steadily improved and have reached promising performances [4,5,6,7,8,9,10,11]. Even numerous commercial software products have become available [12]. Algorithms cannot fully replace the radiologists’ assessments, but using the AI outcomes as assistance for radiologists is an increasingly important topic. AI assistance can be used concurrently (direct access to the AI results) or as a second reader (initial assessment by the radiologist and thereafter access to the AI results). Most published papers on AI algorithms for lung nodule assessment, however, only focus on the standalone performances of the AI systems. Before implementing AI systems in clinical practice, it is important to assess the effects of using the AI system on the performances of radiologists. Some AI-based tools to assist radiologists have been or are currently being implemented in clinical practice; however, the question is whether radiologists and patients really benefit from the use of these algorithms for assessing lung nodules on CT scans.

The aim of this study was to review the effects of using AI assistance for lung nodule detection and malignancy prediction on the performances of radiologists using CT scans. As secondary outcomes, we analyzed the reading time and the provided information on the radiologists’ workflow for using the AI assistance.

## 2. Methods

This systematic review was carried out according to the Preferred Reporting Items for Systematic Reviews and Meta-Analyses (PRISMA) guidelines [13]. We registered this review on PROSPERO (CRD42022331486).

### 2.1. Eligibility Criteria

Studies were eligible for inclusion if (1) they provided performance outcomes of observers both with and without AI-based assistance; (2) AI was used for the detection (finding nodules) or malignancy prediction (distinction between malignant and benign) of lung nodules on CT scans; and (3) the performance was described in terms of sensitivity or Area Under the Curve (AUC), or the sensitivity or AUC could be calculated based on the provided data. Studies were excluded if (1) they did not specify a reference standard (the ground truth with which the radiologists’ assessments were compared); (2) they were unpublished or only published as an abstract; (3) the paper was written in non-English; or (4) they were published before 2017. Studies published between 2017 and 2022 were considered to include adequate up-to-date evidence since the use of AI in lung nodule assessment develops rapidly.

### 2.2. Search Strategy

A single reviewer (LE) performed a comprehensive literature search up to the end of May 2022 from the electronic databases PubMed/MEDLINE, EMBASE and Cochrane Library. The complete searches are documented in Supplementary Materials.

### 2.3. Study Selection

The obtained articles were de-duplicated and selected using a Rayyan database (Rayyan Systems Inc., Cambridge, MA, USA). Two reviewers (LE and KW) independently screened all citations based on the title and abstract. The full texts of potentially eligible studies were subsequently independently assessed according to the predefined inclusion criteria. In addition, the reference lists of the included studies were screened for additional relevant studies. Any disagreements between inclusions were discussed to achieve consensus. If required, a third reviewer (JN) was consulted to determine whether to include the study.

### 2.4. Data Extraction and Synthesis

A single reviewer (LE) extracted data from the included studies, including the population, observers and scan characteristics; the used AI algorithm/tool; the reference standard; and the provided performance metrics. The following performance metrics were collected: the sensitivity, specificity, False Positives (FPs) per scan and AUC. If possible, missing performance metrics were calculated based on specified data. Additionally, if mentioned, we obtained the reporting time and documentation on the workflow for using the AI assistance. All extracted data were inserted in tables. A second reviewer (KW) audited the data extraction. Any disagreements were resolved by discussion.

### 2.5. Quality Assessment

The methodological quality of the studies was evaluated independently by two reviewers (LE and KW) using the Quality Assessment of Diagnostic Accuracy Studies (QUADAS-2) tool. Using this validated tool, the risk of bias and applicability concerns were assessed as low, high or unclear using the signaling questions in the following four domains: patient selection, index test, reference standard, and flow and timing [14]. Discrepancies between reviewers were resolved by consensus.

## 3. Results

### 3.1. Study Selection

**Figure 1:**
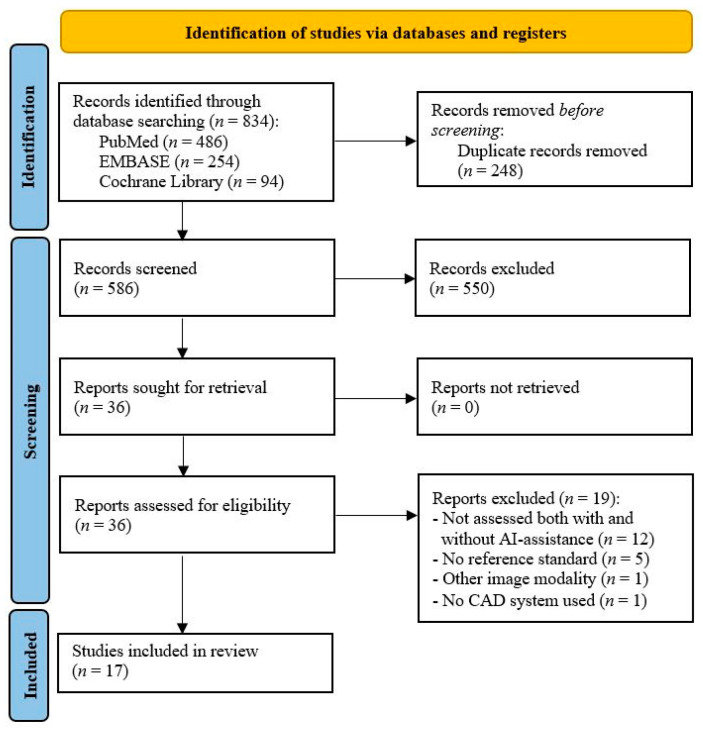
PRISMA flow diagram of the search for eligible studies.

The selection process is presented in the PRISMA flow diagram in Figure 1. Of the 586 unique records, 36 studies were selected to assess at the full-text level. Of these selected studies, 19 were excluded for the following reasons: scans were not assessed by radiologists with and without AI assistance (*n* = 13), the assessments were not compared to a reference standard (*n* = 5), and the imaging modality was not a CT scanner (*n* = 1). As a result, this systematic review included a total of 17 studies.

### 3.2. Study Characteristics

Of the 17 included studies, 13 focused on lung nodule detection and 4 on malignancy prediction. Table 1 and Table 2 provide the characteristics of all studies. Different patient populations were considered, including participants of screening programs (*n* = 6) and patients with a clinically indicated CT scan (*n* = 13). Low-Dose CT (LDCT) scans were used in five studies, Standard-Dose CT (SDCT) scans in nine studies, and both types of scans in three studies. Most studies were single-center studies (*n* = 12). The number of scans that were used for testing the performances ranged from 40 to 2303, with the total number of nodules ranging from 45 to 743. Studies applied different AI algorithms and reference standards and took into account varying nodule sizes and types. In all studies, CT assessments were performed by radiologists. Resident radiologists were involved in seven studies, (chest) physicians in three studies, a chest surgeon in one study and students in two studies.

### 3.3. Quality Assessment

**Figure 2:**
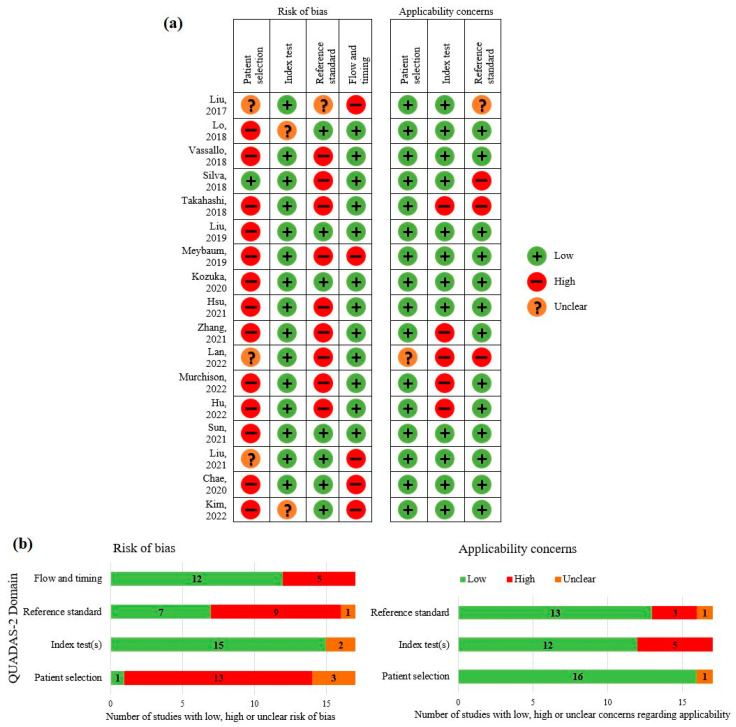
Summarized QUADAS-2 assessments of included studies concerning risk of bias and concerns regarding applicability per study (**a**) and per QUADAS-2 domain (**b**) [15,16,17,18,19,20,21,22,23,24,25,26,27,28,29,30,31].

Figure 2 shows an overview of the judged risks of bias and applicability concerns. All studies had a high risk of bias in any of the four domains, mostly in the domains of patient selection and reference standard. High risks of bias concerning the patient selection were mostly caused by the exclusion of patients with lung diseases. In the reference standard domain, studies were considered to have a high risk of bias mostly due to the formulation of the reference standard with knowledge of the results of the index test. Most concerns about applicability were found in the index test domain and were mostly caused by the fact that the assessments with and without AI were not performed by the same radiologists.

### 3.4. Data Analysis

No meta-analysis was performed due to the great heterogeneity of the studies. This heterogeneity was caused by, amongst others, the varying algorithms, populations, nodule types and sizes, and reference standards.

#### 3.4.1. Detection

The radiologists’ performances on lung nodule detection were reported in the majority of the included papers (13/17) (Table 3). The sensitivity was described in 12 publications and was higher when AI assistance was used in all studies. Differences in sensitivity between reading with and without AI assistance varied from 6.7 to 55.6 percentage points (pps). The specificity was reported by five studies, which mostly demonstrated a lower specificity with AI assistance. By contrast, a higher specificity was reported by the study of Hsu et al. The number of FP findings with and without AI assistance was reported in six papers and was higher with AI assistance, except in the study of Lan et al. The reporting time was described in six papers and was shortened by 11.3 to 30% using the concurrent reading mode (direct access to the AI outcomes), while a second reading (initial assessment by the radiologist and thereafter access to the AI outcomes) extended the reporting time by 11 to 26%. In total, 3 studies computed the AUC, which was approximately 0.1 larger with AI assistance in all studies.

#### 3.4.2. Malignancy Prediction

Malignancy prediction was addressed by four studies (Table 4). Of these studies, three reported the sensitivity and specificity. Compared to without AI assistance, with AI assistance, the sensitivity was higher in two studies (3.8 to 10.5 pps) and lower in one study (5 pps). The specificity was larger with AI assistance in all studies, with the differences between reading with and without AI assistance varying from 2.6 to 42 pps. The AUC was reported by three studies, which all reported a larger AUC (0.02 to 0.07) with AI assistance.

#### 3.4.3. Radiologists’ Workflow

Table 5 gives a summarized overview of the information that the papers provided about the radiologists’ workflow. The functionalities of the workstation used for unassisted reading were described in three of the 17 papers (one briefly). How the nodule position was marked or how scans were scored was described in eight papers (three briefly). Concerning AI-assisted reading, the way of transferring the CT images to the AI tool was described in three papers (one briefly), training for familiarization with the AI tool was described in seven papers (one briefly), how the AI results were shown to radiologists was described in ten papers (four briefly), and what radiologists could do with the AI results was described in eight papers (three briefly). Two papers mentioned how the positions of the marked nodules were compared with the reference standard.

## 4. Discussion

This systematic review provides a structured overview of 17 recent studies on the effects of using AI assistance for the detection and malignancy prediction of lung nodules on CT scans. Concerning detection, studies generally demonstrated that radiologists achieved a higher sensitivity with AI assistance, with only slightly more FPs. Concerning malignancy prediction, generally, both the sensitivity and specificity were higher with AI assistance. The radiologists’ workflows for using the AI assistance were often described in limited detail or were not described at all in the papers.

During a period of more than 5 years, only 17 papers have been published on studies that compared the performances of radiologists with and without AI assistance. Further, in the years before 2017, only a few articles have been published on this topic. The limited number of validation studies in clinical practice might be caused by the gap between the AI developers and the radiologists. A potential explanation is that assessing scans both with and without AI assistance is time-consuming, while being critical to show the added value of an AI tool. Another cause of the limited number of published studies may be publication bias. It is a matter of concern that of the many commercially available AI products for lung nodule assessment [12], only a few have been described in scientific articles in terms of their added effect in clinical practice.

An earlier systematic review, published in 2021 by Li et al., evaluated the effects of AI assistance for lung nodule assessment on CT scans, as well [32]. Even though they applied similar inclusion criteria, only four studies overlapped with the studies that we included. Despite the varying included studies, they found similar results, with generally higher sensitivities and higher AUC values with AI assistance.

### 4.1. Detection and Malignancy Prediction: Performances of Radiologists

Concerning the detection of lung nodules, the generally higher sensitivity with AI assistance indicates a reduced number of overlooked nodules. Potentially malignant nodules might be detected in an earlier stage, improving the chances of an early start of treatment. However, more benign nodules can also be found that require (potentially unnecessary) follow-up. The higher FP detection rate with AI assistance means that more structures that are not nodules are considered in the formulation of the follow-up advice. The extent to which the number of FPs increases, however, seems to be minor.

According to the malignancy prediction, the higher sensitivity, specificity and AUC values with AI assistance demonstrate improved performances in distinguishing malignant from benign nodules. This improved performance can possibly influence the intensity of follow-up and opens possibilities for a more individual risk-based follow-up.

The sizes of the effects of AI assistance on the performances of radiologists differed considerably among studies. These differences might be caused by a variety of factors, such as the quality of the algorithm, the types of patient populations with different nodule types, sizes and prevalence, observers’ expertise and the reference standards. A remarkable finding is, for instance, the exceptional higher sensitivity with AI assistance than without in the study of Silva et al. (34.2% to 88.4%) [18]. This effect can be declared by the poor reference standard, consisting of all detected nodules by either or both AI and the radiologists.

### 4.2. Reading Time

With AI assistance, the reporting time shortened with concurrent readings and increased with second readings. Two of our included studies investigated if concurrent or second readings resulted in better performances (Table 3) [23,27]. The sensitivity was about 2 pps higher with second readings than with concurrent readings. However, in clinical practice, concurrent reading is much more feasible since it saves time, which is valuable because of the full schedules of radiologists.

### 4.3. Radiologists’ Workflow

From the papers, it is impossible to determine how much attention was paid by the studies to the implementation of the AI tool into the radiologists’ workflow since the authors barely provided information about the methods of using the AI assistance and why it was used like that (Table 5). The way of using the AI assistance, however, is very important for the critical step of implementation into clinical practice. AI tools must be easy to use and have to fit seamlessly into the radiologists’ workflow. Many factors may affect whether radiologists adopt the suggestions of the AI algorithm in their assessments, such as the radiologists’ knowledge of the capabilities of the algorithm, their familiarization with the tool and the ways of displaying the algorithms’ results and uncertainty.

### 4.4. Shortcomings in Research on AI Assistance for Lung Nodule Assessment

The potential of using AI assistance for lung nodule assessment has been demonstrated; however, more research must be conducted before it can be widely implemented into the clinic. First, with only four publications in total, research on the effects of AI assistance on lung nodule malignancy prediction is scarce. 

Second, future research should focus on the validation of the AI algorithms and the clinical utility of AI tools. From most of the included papers, it is unclear if the algorithm itself had been extensively validated and which data were used for training. Data of different hospitals may vary because of the different used CT scanners and settings, and therefore, validation with external datasets is essential. Moreover, the applicability of an algorithm may differ between different types of patient populations. Next to the algorithm, the clinical utility of tools has to be validated. In the included studies, the tools were mostly validated on small, selected populations. However, extensive validation is required since assessment performances may vary between different radiologists. Populations must be clinically representative and not biased by selection. 

Third, to assess the actual clinical impact of using the AI assistance, the effects on the follow-up recommendations, which determine a patient’s diagnostic and treatment path, have to be evaluated. Earlier diagnosis of malignant nodules leads to an earlier start of treatment, which increases the chances of patients being cured. Improved diagnostics for benign nodules can result in more appropriate follow-up recommendations, which may prevent unnecessary follow-up scans and biopsies. However, it is important to prevent overdiagnosis since that may cause unnecessary follow-up scans, biopsies, diagnosis- or treatment-related morbidities and anxiety in patients [33]. Adams et al. explored the integration of AI malignancy predictions into the management of lung nodules by a simulation study, in which they up- and downgraded Lung-RADS scores rated by radiologists according to the malignancy prediction of the algorithm. They also analyzed the economic effects and estimated a saving of USD 72 per screened patient [34]. More research has to be performed on the integration of AI into lung nodule management and the cost-effectiveness. 

Moreover, prospective studies have to be conducted to test the added value in clinical practice since the assessments of CT scans and consecutive follow-up recommendations may differ between the lab conditions with retrospective data, as was the case in most studies included in this review, and clinical practice [35,36].

Future studies should focus on the implementation phase and are strongly advised to follow reporting guidelines to standardize the information and metrics that are provided in the papers, enabling better comparison between studies and ensuring transparency. The following guidelines have been developed or are under development: the developmental and exploratory clinical investigations of decision support systems driven by AI (DECIDE-AI), the standards for reporting diagnostic accuracy studies that use AI techniques (STARD-AI) and the transparent reporting of a multivariable prediction model of individual prognosis or diagnosis (TRIPOD-AI) [37,38,39].

### 4.5. Study Limitations

A major limitation of this review is that no meta-analysis could be performed to determine an overall effect because of the heterogeneity of the included studies. Furthermore, comparison was hampered by the fact that the papers provided varying performance metrics. Other limitations are that we included only papers published after 2017 and only English-written papers. 

The evaluation of the quality of the studies according to the QUADAS-2 tool showed a high risk of bias in some domain for all studies, and there were high or unclear concerns about the applicability in some domain in seven studies. These shortcomings may have impaired the reliability of the study results.

## 5. Conclusions

AI assistance for lung nodule assessment holds great promise, as recent studies showed higher sensitivities at the cost of only a slight increase in false positives with AI assistance for the detection, and generally both higher sensitivities and specificities for malignancy prediction. Studies on AI for lung nodule assessment are very heterogeneous, and the methods of using the AI assistance were barely described in the papers. To achieve the added value of AI tools for lung nodule assessment in clinical practice, more research is required on the clinical validation of AI tools, the impact on follow-up recommendations and ways of using AI tools.

## Figures and Tables

**Table 1:** Characteristics of the included studies on the detection of lung nodules.

First Author, Year of Publication, Country	Population	Exclusion Criteria	Algorithm/ Software	Use of Assistance	CT Scans	N Scans	N Scans with Nodules	N Nodules	Nodule Sizes	Nodule Types	Reference Standard (Years of Experience)	N Observers (Years of Experience)
Liu, 2017 [15], China	Participants of screening	-	Sparse non-negative matrix factorization model	SR	SDCT - Single-center	180	80	96	4–10 mm	Various types	Biopsy, resection or consensus by radiologists	6 (2 resident radiologists (<5), 2 secondary chest radiologists (8–12), 2 senior chest radiologists (>15))
Lo, 2018 [16], Unites States	Participants of screening	-	VIS/CADe	CR	LDCT No contrast Multi-center	324	108	178	≥5 mm	Solid, part-solid, GGO	Detected by at least 2/3 radiologists	12 radiologists (6–26)
Vassallo, 2018 [17], Italy	Patients with an extra-thoracic primary tumor	Clinical conditions that may mimic or hide lung nodules >10 nodules	M5L lung CAD on-demand	SR	SDCT With and without contrast Single-center	225 (28 with contrast)	75	215	≥3 mm	Solid, part-solid, sub-solid, calcified	Consensus by 2 radiologists (>15) who assessed all nodules detected by at least 1 radiologist or/and CAD	3 (1 resident radiologist (3), 2 radiologists (20, 35))
Silva, 2018 [18], Italy	Participants of screening	-	CIRRUS Lung screening (prototype version of Veolity)	CR	LDCT No contrast Multi-center	2303	155	194	≥5 mm	Sub-solid (including non-solid, part-solid)	Detected by either or both radiologists or CAD	AI-assisted: consensus by 2 radiologists (8, 11) Radiologists only: 1 out of 7 radiologists (4–20)
Takahashi, 2018 [19], United States	Patients with clinically indicated CT scan	Clinical conditions that may hide or obscure lung nodules >10 nodules	Syngo.CT Lung CAD (Siemens Health Care)	SR	Ultra LDCT No contrast Single-center	55	30	45	≥5 mm	Solid, part-solid, GGN	Assessed by 1 radiologist (>20) using SDCT (acquired consecutively)	2 out of 4 radiologists (5–21)
Liu, 2019 [20], China	Patients who underwent a clinical scan and participants for screening	No clinical report Postoperative Lung diseases Confident ground truth annotation impossible Incomplete coverage of lungs Motion artifacts No compliance with DICOM	InferRead CT Lung (Infervision)	CR	LDCT and SDCT - Multi-center	123 for per-nodule analysis, 148 for per-patient analysis	-	-	>3 mm	Solid	Assessment by 2 radiologists (±10) with access to original radiology report, differences checked by 3rd radiologist (±10)	2 radiologists (±10)
Meybaum, 2019 [21], Germany	Patients who underwent surgery for lung metastases	Chest CT scan older than 8 weeks before surgery Slice thickness > 2 mm, reconstruction increment > 1 mm	LMS 6.0, Median Technologies, Valbonne, France	SR	SDCT - Single-center	95	-	646	-	-	Surgical findings during pulmonary metastasectomy	1 out of 4 radiologists (6–20)
Kozuka, 2020 [22], Japan	Patients with suspected lung cancer	Severe postoperative complications Pneumonia, diffuse lung disease, massive pleural effusion/ atelectasis Inappropriate image quality	InferRead CT Lung (Infervision)	CR	SDCT No contrast Single-center	117	111	743	≥3 mm	Solid, part-solid, calcified, GGN	Assessment by 2 radiologists, differences checked by 3rd radiologist (6, 12, 26)	2 radiologists (1, 5)
Hsu, 2021 [23], Taiwan	Patients who underwent a CT scan with proved small pulmonary nodules (≤10 mm), stable nodules for at least 2 years, incidental small nodules on screening	Clinical conditions that may mimic or hide lung nodules >10 nodules	ClearRead CT	CR and SR	LDCT and SDCT No contrast Single-center	150 (57 LDCT, 93 SDCT)	98	340	≤10 mm	Solid, part-solid, GGN	Consensus by 2 radiologists (>15), first assessed without pre-knowledge, subsequently reviewed all nodules detected by any radiologist or/and AI	6 (3 resident radiologists (1–2), 3 radiologists (5, 10, 25))
Zhang, 2021 [24], China	Asymptomatic participants of screening	No or incomplete clinical report History of lung surgery Confident ground truth annotation impossible Incomplete coverage of lungs Motion artifacts	InferRead CT Lung (Infervision)	CR	LDCT No contrast Single-center	860	250 solid; 13 part-solid; 111 non-solid (at least 1 nodule of that type)	-	≥3 mm	Solid, part-solid, non-solid	Assessment by 2 radiologists (20, 31) who assessed all scans with pre-knowledge of nodules detected by radiologists or AI	AI-assisted: Consensus by a resident radiologist (5) supervised by a radiologist (20) Radiologists only: 1/14 resident radiologists (2–5) supervised by 1/15 radiologists (10–30)
Lan, 2022 [25], Taiwan	Patients who underwent a CT scan	-	ResNet 18	CR	SDCT - Single-center	60	-	266	-	Solid, part-solid, GGO	Detected by AI and at least 3/5 doctors	5 (2 assessed with AI, 3 without AI): 3 chest physicians, 1 chest surgeon, 1 radiologist, (all >10 years of experience in reading chest CT scans)
Murchison, 2022 [26], United Kingdom	(Previous) smokers and/or radiological evidence of pulmonary emphysema	Presence of diffuse pulmonary disease in radiology report Widespread abnormalities, such as interstitial lung disease Slice thickness > 3 mm	Veye Lung Nodules (Aidence)	CR	SDCT With and without contrast Single-center	273 (22 with contrast)	-	269	5–30 mm	Solid, sub-solid	Detected by both radiologists or detected by 1 radiologists and confirmed by third radiologist	1 out of 2 radiologists (≥9)
Hu, 2022 [27], China	Patients with an extra-thoracic primary tumor	Severe pulmonary fibrosis, severe emphysema, extensive pulmonary infection, tuberculosis/sarcoidosis, massive pleural effusion	InferRead CT Lung (Infervision)	CR and SR	LDCT No contrast Single-center	117	-	650	-	Solid, sub-solid calcified	Consensus by 2 radiologists (>20) who assessed all scans with access to CAD outcomes	9 (3 radiologists (5–10), 3 resident radiologists (2–3), 3 interns (<1))

‘-’ indicates being unmentioned in the study. Abbreviations: AI, Artificial Intelligence; CAD, Computer-Aided Detection/Diagnosis; CR, Concurrent Reading; GGO, Ground-Glass Opacity; GGN, Ground-Glass Nodule; LDCT, Low-Dose CT; SDCT, Standard-Dose CT; SR, Second Reading.

**Table 2:** Characteristics of the included studies on the malignancy prediction of lung nodules.

First Author, Year of Publication, Country	Population	Exclusion Criteria	Algorithm/ Software	Use of Assistance	CT Scans	N Scans	N Scans with Nodules	N Nodules	Nodule Sizes	Nodule Types	Assessment Observers	Output AI	Reference Standard (Years of Experience)	N Observers (Years of Experience)
Sun, 2021 [28], China	Patients with a primary intrapulmonary lesion	Clearly benign diagnosis History of malignancy Lesions with calcification Obvious artefacts on CT	ResNet-101	CR	SDCT - Single-center	47	47	-	>8 mm	Solid intermediate solitary pulmonary nodules	Benign or malignant	Both benign and malignant probability in percentages	Histopathologic confirmed	Consensus by 2 radiologists (4, 9) and in cases of discrepancies a third radiologist (>29)
Liu, 2021 [29], China	Patients with lung nodules	-	ResNet-50	SR	SDCT - Multi-center	-	-	168 (112 malignant, 56 benign)	6–30 mm	-	5 categories	Number between 0 and 1	Histopathologic confirmed or benign based on stability on CT during 2 years or disappeared	6 (4 radiologists (8, 12, and two >15), 2 resident radiologists (<5))
Chae, 2020 [30], South Korea	Patients with difficult-to-diagnose lung nodules	Active pulmonary lesions (such as pneumonia or pulmonary tuberculosis)	CT-lungNET	SR	SDCT No contrast Single-center	60	60	60 (30 malignant, 30 benign)	5–20 mm	-	4 categories	Percentages	Histopathologic confirmed or benign based on stability on CT during >1 year	8 (2 third-year medical students, 2 physicians (non-radiology) (1), 2 resident radiologists (2), 2 radiologists (3, 5))
Kim, 2022 [31], United Kingdom	Patients with indeterminate lung nodules	>2.5 mm pixel spacing >5 IPNs IPNs < 5 mm in largest diameter	Virtual Nodule Clinic (Optellum)	SR	LDCT (*n* = 124) and SDCT (*n* = 176) No contrast Multi-center	300	300 (150 malignant, 150 benign)	300 (150 malignant, 150 benign)	5–30 mm	Solid, mixed, part-solid	Score between 0 and 100	Number between 1 and 10 (decile scale)	Histopathologic confirmed or benign based on stability on CT during 2 years or disappeared	12 (6 pulmonologists (2 in thoracic oncology), 6 radiologists (2 in thoracic radiology))

‘-’ indicates being unmentioned in the study. Abbreviations: AI, Artificial Intelligence; CR, Concurrent Reading; IPN, Indeterminate Pulmonary Nodule; LDCT, Low-Dose CT; SDCT, Standard-Dose CT; SR, Second Reading.

**Table 3:** Performance outcomes of studies focusing on the detection of lung nodules. When mentioned in the study, the sensitivity, specificity, AUC, number of FPs and the reporting time from AI-only, observers only and AI-assisted reading were provided.

First Author, Year of Publication	Per-Nodule/Per-Patient (Mean) Sensitivity 95% CI TP/(TP + FN)			Per-Patient (Mean) Specificity 95% CI TN/(TN + FP)			(Mean) AUC 95% CI		Mean FP Per Scan			Reporting Time Mean Time Per Scan ± SD		
	AI	Observers	AI-Assisted	AI	Observers	AI-Assisted	Observers	AI-Assisted	AI	Observers	AI-Assisted	Observers	AI-Assisted	Difference
Liu, 2017 [15]	Per-nodule			-	-	-	-	-	0.09	0.016	-	-	-	-
	92.7% 89/96	87.3% 503/576	96.9% 558/576											
Lo, 2018 [16]	Per-nodule			-	89.9% 85.9–93.9% 194/216	84.4% 80.4–88.4% 182/216	0.584 0.518–0.650	0.692 0.626–0.758	0.58	0.17	0.28	132.3 s	98.0 s	−26%
	82.0% 146/178	60.1% 53.5–66.7% 107/178	72.5% 65.9–79.1% 129/178											
Vassallo, 2018 [17]	Per-nodule			-	85% 71–79% 382/450	82% 79–86% 369/450	-	-	3.8	0.13	0.16	296 ± 80 s	329 ± 83 s	+11% *
	85% 82–91% 183/215	65% 61–69% 421/645	88% 86–91% 570/645											
	Per-patient													
	-	75% 71–79% 169/225	82% 79–86% 186/225											
Silva, 2018 [18]	Per-nodule			-	-	-	-	-	-	-	0.26	-	-	-
	-	37.1% 72/194	90.2% 175/194											
	Per-patient													
	-	34.2% 53/155	88.4% 137/155											
Takahashi, 2018 [19]	Per-nodule			-	-	-	-	-	2	0.45	-	-	-	-
	71% 57–82% 32/45	~60%	70.8% 63/89											
Liu, 2019 [20]	Per-nodule			-	-	-	Per-patient		-	-	-	±15 min	±5–10 min	-
	-	-	Improved, shown by radar plot				0.660	0.778						
Meybaum, 2019 [21]	Per-nodule			-	-	-	-	-	-	-	-	-	-	-
	53.6% 49.7–57.4% 346/646	67.5% 63.9–71.1% 436/646	77.9% 74.7–81.1% 503/646											
Kozuka, 2020 [22]	Per-nodule			83.3% 35.9–99.6% 5/6	91.7% 61.5–99.8% 11/12	83.3% 51.6–97.9% 10/12	-	-	3.25	1.11	2.97	3.1 min	2.8 min	−11.3%
	70.3% 66.8–73.5% 522/743	20.9% 18.8–23.0% 310/1486	38.0% 35.5–40.5% 564/1486											
	Per-patient													
	95.5% 89.8–98.5% 106/111	68% 61.4–74.1% 151/222	85.1% 79.8–89.5% 189/222											
Hsu, 2021 [23]	Per-nodule			87% 45/52	80% 78–81% 250/312	CR: 83% 82–85% 259/312 SR: 84% 82–85% 262/312	0.72 0.70–0.74	CR: 0.82 0.80–0.83 SR: 0.83 0.81–0.85	0.67 (diameter threshold > 5 mm)	-	-	CR: 156 ± 34 s SR: 156 ± 34 s	CR: 124 ± 25 s SR: 197 ± 46 s	CR: −21% SR: +26% *
	86% 291/340	64% 62–66% 1306/2040	CR: 80% 79–82% 1632/2040 SR: 82% 80–84% 1673/2040											
Zhang, 2021 [24]	Per-patient			-	100%	~99%	-	-	-	-	-	-	-	-
	-	43.3% 162/374	98.9% 370/374											
Lan, 2022 [25]	Per-nodule			-	-	-	-	-	-	0.634	0.122	-	-	-
	-	63.1% 52.0–74.1% 504/798	69.8% 50.9–88.6% 371/532											
Murchison, 2022 [26]	Per-nodule			-	-	-	-	-	-	0.11	0.16	-	-	-
	-	71.9% 66.0–77.0% 193/269	80.3% 75.2–85.0% 216/269											
Hu, 2022 [27]	Per-nodule			-	-	-	-	-	-	SDCT: 1.90 LDCT: 1.76	SDCT: 2.89 LDCT: 2.63	-	-	-
	-	SDCT: 45.35% 2694/5940 LDCT: 42.11% 2463/5850	SDCT: 57.77% 3432/5940 LDCT: 56.70% 3317/5850											
	-	44.27% 1197/2704	CR: 64.76% 1751/2704 SR: 66.86% 1808/2704	-	-	-	-	-	-	2.67	CR: 4.09 SR: 4.11	CR: 235 ± 162 s SR: 235 ± 162 s	CR: 165 ± 133 s SR: 294 ± 153 s	CR: −30% SR: +25% *

‘-’ indicates being unmentioned in the study. * AI used as SR. Abbreviations: AI, Artificial Intelligence; AUC, Area Under the Curve; CI, Confidence Interval; CR, Concurrent Reading; FN, False Negatives; FP, False Positives; LDCT, Low-Dose CT; SDCT, Standard-Dose CT; SR, Second Reading; TN, True Negatives; TP, True Positives.

**Table 4:** Performance outcomes of studies focusing on the malignancy prediction of lung nodules. When mentioned in the study, the sensitivity, specificity and AUC from AI-only, observers only and AI-assisted reading were provided.

First Author, Year of Publication	Malignancy Risk Threshold	Per-Nodule								
		(Mean) Sensitivity 95% CI TP/(TP + FN)			(Mean) Specificity 95% CI TN/(TN + FP)			(Mean) AUC 95% CI		
		AI	Observers	AI-Assisted	AI	Observers	AI-Assisted	Observers	AI-Assisted	Difference
Sun, 2021 [28]	-	86%	82%	89%	79%	42%	84%	0.91	-	-
		73–93%	69–90%	77–95%	65–88%	29–56%	71–92%	0.83–0.99		
Liu, 2021 [29]	58% (Youden Index)	93.8%	-	-	83.9%	-	-	-	0.913	0.938
Chae, 2020 [30]	-	60%	70%	65%	87%	69%	85%	0.85	~0.72	~0.79
								0.74–0.93		
Kim, 2022 [31]	5%	-	94.1%	97.9%	-	37.4%	42.3%	-	0.82	0.89
			90.8–97.4%	96.0–99.7%		27.2–47.6%	31.3–53.3%			
			1693/1800	1762/1800		674/1800	761/1800			
	65%	-	52.6%	63.1%	-	87.3%	89.9%		0.77–0.86	0.85–0.92
			41.8–63.3%	53.7–72.5%		81.0–93.6%	83.3–96.6%			
			946/1800	1136/1800		1572/1800	1619/1800			

‘-’ indicates being unmentioned in the study. Abbreviations: AI, Artificial Intelligence; AUC, Area Under the Curve; CI, Confidence Interval; FN, False Negatives; FP, False Positives; TN, True Negatives; TP, True Positives.

**Table 5:** Provided information on the radiologists’ workflow concerning unassisted reading, AI-assisted reading and the comparison with the reference standard.

First Author, Year of Publication	Unassisted Reading			AI-Assisted Reading					Comparison with Reference Standard	
	Provided Information	Functionalities Workstation	How Nodule Positions were Marked/Scans were Scored	Provided Information	How Images were Transferred to the AI Tool	Training for Familiarization with the AI Tool	How AI Results were Shown	What Radiologists Could do with the AI Results	Provided Information	Information Provided
Liu, 2017 [15]	Observers interpreted the original CT images using the PACS workstation. Observers marked the detected nodule on the images using a drawing tool internally installed in the workstation.	N	B	Before the observation, we introduced the CAD system to the observers to make sure they can use it. All observers were told the accuracy of the CAD technique before the performance study. The CAD output image was displayed on a monitor. The observers made a refined decision after taking CAD output into consideration. However, if they make a change, they must record it.	N	B	B	N	-	N
Lo, 2018 [16]	A workstation with standard interpretation functions (i.e., zoom, pan, magnified glass, maximum intensity projection, sagittal or coronal viewing, lung or soft-tissue window setting, window or level control and other functions) was used. Radiologists marked the center of each nodule.	Y	B	Fifteen training cases were performed to familiarize with the workstation and its interpretation functions. Detected nodules by AI were marked with a score that indicated the likelihood that a nodule was present. If the score was greater than a selected threshold value, the nodules were shown as boundary contours on both the standard CT scan (left) and vessel-suppressed image (right). These images were coupled. Nodule’s density, maximum and minimum diameters, and volume were provided. The same process as during unassisted reading was performed.	N	Y	Y	N	If a mark of the radiologist was within the ellipsoid range of the nodule defined by the reference standard, the mark was considered as TP, otherwise as FP.	Y
Vassallo, 2018 [17]	CT images were stored on picture archiving and communication system (PACS). Maximum-intensity projection volume-rendering technique was used. The radiologists searched freely for lesions and measured the longest diameter of each nodule using a lung window on the plane where the lesion was more conspicuous, provided the nodule coordinates. All information was stored on an online web-form.	B	Y	The AI-detected nodules became accessible through a dedicated web-form. Radiologists could classify unmatched findings as FP, TP or irrelevant (<3 mm).	B	N	N	Y	If the Euclidean distance between the 3D centers of the mark of the radiologist and the AI-detected nodule was within the nodule diameter, the mark was considered TP. This was an automatic process.	Y
Silva, 2018 [18]	-	N	N	Radiologists reviewed the marks of the AI-detected nodules by selecting marks reflecting SSNs and discarding FPs.	N	N	N	Y	-	N
Takahashi, 2018 [19]	Radiologists marked nodules using a specially configured workstation (Mayo Biomedical Imaging Resource).	N	N	The CAD system is a fully automated computer-assisted tool that can identify pulmonary nodules regardless of density. The CAD markings were shown on the CT images in addition to their corresponding unassisted detections. They characterized each CAD marking as TP or FP.	N	N	B	Y	-	N
Liu, 2019 [20]	Radiologists marked nodules by square bounding boxes with the nodule at the center.	N	Y	Output of the model was the detection marked with a square bounding box. The nodule type and the model’s confidence in its prediction were also included. Radiologists used the AI assistance and reported the nodule type and confidence level.	N	N	Y	N	-	N
Meybaum, 2019 [21]	Radiologists used the user interface of the CAD system for CT reading, which provides original images and sliding thin-slab maximum-intensity projection. They labeled all nodules and made a screenshot of each nodule.	B	B	The CT images were transferred to the CAD system, and the CAD analysis was sent to the PACS system. The CAD findings were given. TP findings were labeled, and FPs were rejected. The total number of nodules per CT scan was recorded in a comment field. The radiologist filled out a study documentation sheet containing lesion label, lung segment, lesion size and lung scheme in three views, on which the radiologist marked the approximate position of all lesions.	Y	N	N	Y	-	N
Kozuka, 2020 [22]	Radiologists marked each nodule using a workstation with general interpretation functions.	B	N	The CAD system had functions to display marks, density, major axis and the volume of the detected nodules. Object bounding boxes and scores were provided.	N	N	Y	N	-	N
Hsu, 2021 [23]	-	N	N	Radiologists were trained in how to operate the procedures with several training images. The displays of the CAD results were combined to make the final decision. The CAD system renders a boundary contour on detected nodules, which is classified as actionable and provides a report table with nodule density, maximum and minimum diameters, and volume to the radiologists.	N	Y	Y	N	-	N
Zhang, 2021 [24]	-	N	N	The CT images were automatically transmitted to the AI server for segmentation and detection of lung nodules. Radiologists read the results on their image reading terminals. The system automatically depicts a suspected nodule with a bounding box and reveals its characteristics, including its components (solid/part-solid/non-solid), diameter and volume.	Y	N	Y	N	-	N
Lan, 2022 [25]	-	N	N	-	N	N	N	N	-	N
Murchison, 2022 [26]	Actionable nodules were marked manually.	N	N	Radiologists received training on the annotation tasks and annotation tool, with written instructions available throughout. Radiologists classified a CAD prompt as either TP or FP. Any actionable nodules identified on aided scans, which had not been detected by CAD, were also recorded.	N	Y	N	Y	-	N
Hu, 2022 [27]	-	N	N	Radiologists were trained on the CAD system. Radiologists were allowed to adjust the window level, zoom in and out, invert gray-scale and use maximum-intensity projection thick-slab images. Each by AI-identified nodule was marked on the image showing the nodule’s largest diameter.	N	Y	Y	N	-	N
Sun, 2021 [28]	-	N	N	Response heat maps were shown, in which red areas indicate that the model mainly extracted diagnostic characteristics from the region, while blue areas indicate that less discriminative features were found in that region.	N	N	B	N	-	N
Liu, 2021 [29]	Radiologists observed the CT scans with two clinical parameters (age and sex) to rate a diagnostic score. According to the likelihood of malignancy, the diagnosis score ranged from 1 to 5 (highly unlikely, moderately unlikely, indeterminate, moderately suspicious, highly suspicious). The nodules were indicated with red arrows. Radiologists could scroll through the slices, adjust the width and window level, and use the zoom function.	Y	Y	Radiologists were trained on the CAD system, with two training cases to learn how to use the system and learn the rating method. They were introduced to the performance of the CAD system. After initial suspicion rating, the CAD output was displayed on the monitor. Radiologists could update their suspicion.	N	Y	N	B	-	N
Chae, 2020 [30]	Radiologists reviewed the CT scans with the given positions of the centers of the nodules. They interpreted the probability of the malignancy of each nodule on a four-point scale (highly suggestive of benign nature (<25%), more likely benign than malignant (25–50%), more likely malignant than benign (50–75%), highly suggestive of malignant nature (>75%)).	N	Y	Each reviewer reclassified the previously determined category after considering the malignancy prediction rate of the AI system.	N	N	N	B	-	N
Kim, 2022 [31]	Radiologists loaded each of the scans into the software, which highlighted the nodule of interest. They scrolled through the entire set of images with axial views. They estimated the malignancy risk on a 100-point scale and selected a management recommendation (no action, CT follow-up after more than 6 months, CT follow-up after 6 weeks to 6 months, immediate imaging follow-up, nonsurgical biopsy or surgical resection).	N	Y	Radiologists had 1 hour of training with the CAD system and evaluated 17 example cases. The lung cancer prediction score was displayed, and readers were asked to provide an updated risk estimate and management recommendation.	N	Y	B	B	-	N

‘-’ indicates being unmentioned in the study. Y ‘Yes’, described in the paper. B ‘Briefly’, only briefly described in the paper. N ‘No’, not described in the paper. Abbreviations: AI, Artificial Intelligence; CAD, Computer-Aided Detection/Diagnosis; FP, False Positives; TP, True Positives.

## Data Availability

Data sharing is not applicable.

## Supplementary Materials

The following supporting information can be downloaded at: https://www.mdpi.com/article/10.3390/jcm12103536/s1.
