# Supplementary material for: The Effects of Artificial Intelligence Assistance on the Radiologists’ Assessment of Lung Nodules on CT Scans: A Systematic Review
Source: J Clin Med. 2023 May 18;12(10):3536. doi: 10.3390/jcm12103536 (PMC10219568; doi:10.3390/jcm12103536)
Supplement: Supplementary file 1 [file jcm-12-03536-s001.zip › jcm-2315681-supplementary.pdf]

Supplementary

Pubmed
Date of search: 31 May 2022
Publication date restriction: 2017-2022
Search query: ((((("Lung Neoplasms"[Mesh]) OR (Pulmonary Neoplasm*[Title/Abstract] OR Lung Neoplasm*[Title/Abstract] OR Lung Cancer*[Title/Abstract] OR Pulmonary Cancer*[Title/Abstract] OR Cancer of the Lung*[Title/Abstract] OR Cancer of Lung*[Title/Abstract] OR Lung nodule*[Title/Abstract] OR Pulmonary nodule*[Title/Abstract] OR Lung tumor*[Title/Abstract] OR Lung tumour*[Title/Abstract] OR Lung carninoma*[Title/Abstract])) AND (("Tomography, X-Ray Computed"[Mesh]) OR (Computed Tomography[Title/Abstract] OR Computed X Ray Tomography[Title/Abstract] OR X-Ray Computer Assisted Tomography[Title/Abstract] OR X Ray Computer Assisted Tomography[Title/Abstract] OR X-Ray Computerized Tomography[Title/Abstract] OR Tomodensitometry[Title/Abstract] OR Computed X-Ray Tomography[Title/Abstract] OR X-Ray CAT Scan*[Title/Abstract] OR Electron Beam Tomography[Title/Abstract] OR X-Ray Computerized Axial Tomography[Title/Abstract] OR X Ray Computerized Axial Tomography[Title/Abstract] OR CT scan*[Title/Abstract] OR CT-scan*[Title/Abstract]))) AND (((("Artificial Intelligence"[Mesh]) OR "Deep Learning"[Mesh]) OR "Machine Learning"[Mesh]) OR (Computational Intelligence[Title/Abstract] OR Machine Intelligence[Title/Abstract] OR Computer Reasoning[Title/Abstract] OR Artificial Intelligence[Title/Abstract] OR Computer Vision System*[Title/Abstract] OR Knowledge Acquisition[Title/Abstract] OR Knowledge Representation*[Title/Abstract] OR Hierarchical Learning[Title/Abstract] OR Transfer Learning[Title/Abstract] OR Neural network*[Title/Abstract] OR Machine learning[Title/Abstract] OR Deep learning[Title/Abstract] OR Computer aid*[Title/Abstract] OR Computer-aid*[Title/Abstract] OR Computer assist*[Title/Abstract] OR Computer-assist*[Title/Abstract] OR CAD software[Title/Abstract] OR CAD system*[Title/Abstract] OR Convolutional[Title/Abstract] OR Algorithm*[Title/Abstract] OR Automat*[Title/Abstract]))) AND (((("Early Detection of Cancer"[Mesh]) OR "Risk Assessment"[Mesh]) OR "Classification"[Mesh]) OR (Systematics[Title/Abstract] OR Taxonom*[Title/Abstract] OR Classif*[Title/Abstract] OR Detect*[Title/Abstract] OR Identif*[Title/Abstract] OR Characteri*[Title/Abstract] OR Assess*[Title/Abstract] OR Categori*[Title/Abstract] OR Screen*[Title/Abstract]))) AND ((("Radiologists"[Mesh]) OR "Physicians"[Mesh]) OR (Radiologist*[Title/Abstract] OR Physician*[Title/Abstract] OR Human assessment*[Title/Abstract] OR Observer*[Title/Abstract] OR Clinician*[Title/Abstract] OR Clinical evaluation*[Title/Abstract] OR Concurrent read*[Title/Abstract] OR Concurrent-read*[Title/Abstract] OR Second read*[Title/Abstract] OR Second-read*[Title/Abstract] OR First read*[Title/Abstract] OR First-read*[Title/Abstract] OR Assisted[Title/Abstract] OR CAD-assisted[Title/Abstract] OR Technician*[Title/Abstract] OR AI-assist*[Title/Abstract] OR AI assist*[Title/Abstract] OR Human expert*[Title/Abstract] OR Reviewer*[Title/Abstract] OR Doctor*[Title/Abstract] OR Reader*[Title/Abstract]))
Items found: 486

Embase
Date of search: 31 May 2022
Publication date restriction: 2017-2022
Search query: ('**pulmonary neoplasm***':ti,ab,kw OR '**lung neoplasm***':ti,ab,kw OR '**lung cancer***':ti,ab,kw OR '**pulmonary cancer***':ti,ab,kw OR '**cancer of the lung***':ti,ab,kw OR '**cancer of lung***':ti,ab,kw OR '**lung nodule***':ti,ab,kw OR '**pulmonary nodule***':ti,ab,kw OR '**lung tumor***':ti,ab,kw OR '**lung tumour***':ti,ab,kw OR '**lung carninoma***':ti,ab,kw) AND ('**computed tomography**':ti,ab,kw OR '**computed x ray tomography**':ti,ab,kw OR '**x-ray computer assisted tomography**':ti,ab,kw OR '**x ray computer assisted tomography**':ti,ab,kw OR '**x-ray computerized tomography**':ti,ab,kw OR **tomodensitometry**:ti,ab,kw OR '**computed x-ray tomography**':ti,ab,kw

OR '**x-ray cat scan***':ti,ab,kw OR '**electron beam tomography**':ti,ab,kw OR '**x-ray computerized axial tomography**':ti,ab,kw OR '**x ray computerized axial tomography**':ti,ab,kw OR '**ct scan***':ti,ab,kw) AND ('**computational intelligence**':ti,ab,kw OR '**machine intelligence**':ti,ab,kw OR '**computer reasoning**':ti,ab,kw OR '**artificial intelligence**':ti,ab,kw OR '**computer vision system***':ti,ab,kw OR '**knowledge acquisition**':ti,ab,kw OR '**knowledge representation***':ti,ab,kw OR '**hierarchical learning**':ti,ab,kw OR '**transfer learning**':ti,ab,kw OR '**neural network***':ti,ab,kw OR '**machine learning**':ti,ab,kw OR '**deep learning**':ti,ab,kw OR '**computer aid***':ti,ab,kw OR '**computer assist*or computer-assist***':ti,ab,kw OR '**cad software**':ti,ab,kw OR '**cad system***':ti,ab,kw OR **convolutional**:ti,ab,kw OR **algorithm***:ti,ab,kw OR **automat***:ti,ab,kw) AND (**systematics**:ti,ab,kw OR **taxonom***:ti,ab,kw OR **classif***:ti,ab,kw OR **detect***:ti,ab,kw OR **identif***:ti,ab,kw OR **characteri***:ti,ab,kw OR **assess***:ti,ab,kw OR **categori***:ti,ab,kw OR **screen***:ti,ab,kw) AND (**radiologist***:ti,ab,kw OR **physician***:ti,ab,kw OR '**human assessment***':ti,ab,kw OR **observer***:ti,ab,kw OR **clinician***:ti,ab,kw OR '**clinical evaluation***':ti,ab,kw OR '**concurrent read***':ti,ab,kw OR '**second read***':ti,ab,kw OR '**first read***':ti,ab,kw OR **assisted**:ti,ab,kw OR '**cad assisted**':ti,ab,kw OR **technician***:ti,ab,kw OR '**ai assist***':ti,ab,kw OR '**human expert***':ti,ab,kw OR **reviewer***:ti,ab,kw OR **doctor***:ti,ab,kw OR **reader***:ti,ab,kw) AND [2017-2022]/py AND [embase]/lim AND [english]/lim AND ([article]/lim OR [conference paper]/lim OR [data papers]/lim)
Items found: 254

<u>**Cochrane**</u>
Date of search: 31 May 2022
Publication date restriction: 2017-2022
Search query:

ID	Search	Hits
#1	MeSH descriptor: [Lung Neoplasms] explode all trees	8561
#2	(Pulmonary Neoplasm* OR Lung Neoplasm* OR Lung Cancer* OR Pulmonary Cancer* OR Cancer of the Lung* OR Cancer of Lung* OR Lung nodule* OR Pulmonary nodule* OR Lung tumor* OR Lung tumour* OR Lung carninoma*):ti,ab,kw	31575
#3	MeSH descriptor: [Tomography, X-Ray Computed] explode all trees	5514
#4	(Computed Tomography OR Computed X Ray Tomography OR X-Ray Computer Assisted Tomography OR X Ray Computer Assisted Tomography OR X-Ray Computerized Tomography OR Tomodensitometry OR Computed X-Ray Tomography OR X-Ray CAT Scan* OR Electron Beam Tomography OR X-Ray Computerized Axial Tomography OR X Ray Computerized Axial Tomography OR CT scan* OR CT-scan*):ti,ab,kw	24654
#5	MeSH descriptor: [Artificial Intelligence] explode all trees	1408
#6	MeSH descriptor: [Deep Learning] explode all trees	54
#7	MeSH descriptor: [Machine Learning] explode all trees	234
#8	(Computational Intelligence OR Machine Intelligence OR Computer Reasoning OR Artificial Intelligence OR Computer Vision System* OR Knowledge Acquisition OR Knowledge Representation* OR Hierarchical Learning OR Transfer Learning OR Neural network* OR Machine learning OR Deep learning OR Computer aid* OR Computer-aid* OR Computer assist*OR Computer-assist* OR CAD software OR CAD system* OR Convolutional OR Algorithm* OR Automat*):ti,ab,kw	39808
#9	MeSH descriptor: [Early Detection of Cancer] explode all trees	1462
#10	MeSH descriptor: [Risk Assessment] explode all trees	9440
#11	MeSH descriptor: [Classification] explode all trees	137

#12	(Systematics OR Taxonom* OR Classif* OR Detect* OR Identif* OR Characteri* OR Assess* OR Categori* OR Screen*):ti,ab,kw	810871
#13	MeSH descriptor: [Radiologists] explode all trees	22
#14	MeSH descriptor: [Physicians] explode all trees	2401
#15	(Radiologist* OR Physician* OR Human assessment* OR Observer* OR Clinician* OR Clinical evaluation* OR Concurrent read* OR Concurrent-read* OR Second read* OR Second-read* OR First read* OR First-read* OR Assisted OR CAD-assisted OR Technician* OR AI-assist* OR AI assist* OR Human expert* OR Reviewer* OR Doctor* OR Reader*):ti,ab,kw	378413
#16	#1 OR #2	31751
#17	#3 OR #4	24722
#18	#5 OR #6 OR #7 OR #8	40545
#19	#9 OR #10 OR #11 OR #12	810910
#20	#13 OR #14 OR #15	378692
#21	#16 AND #17 AND #18 AND #19 AND #20	94

Items found: 94
